# The first complete chloroplast genome of *Drynaria acuminata* (Polypodiaceae), a local rare fern species

**DOI:** 10.1080/23802359.2020.1860709

**Published:** 2021-01-27

**Authors:** Kaikai Wang, Hongmei Liu

**Affiliations:** a Xishuangbanna Tropical Botanical Garden, Chinese Academy of Sciences, Menglun, China; b University of Chinese Academy of Sciences, Beijing, China

**Keywords:** Local rare fern, phylogeography, Polypodiaceae

## Abstract

The complete chloroplast genome of a local rare fern species *Drynaria acuminata* was sequenced. The genome has a length of 151,591 bp with 40.8% GC content, with in total 131 genes were annotated, including 88 protein genes, 35 tRNAs, and 8 rRNAs. This work provides basic information for its phylogeographical and conservation research.

*Drynaria acuminata* (Willdenow) C. V. Morton is an epilithic fern belonging to the subfamily Drynarioideae of Polypodiaceae (PPG I 2016). This species is rather widespread in Indonesia, Malaysia, and the Philippines with only a few occurrences in Cambodia, Laos, Thailand, and Vietnam recorded whilst it was only recorded to occur in China till 1990 (Li 1990). The only Chinese location is in Xishuangbanna, Yunnan and is considered to be endangered by deforestation and loss of habitat. Thus, the species was included into the Red List of China Higher Plants (Qin et al. 2017). Based on the herbarium record, *D. acuminata* is considered here as a plant species with extremely small populations (PSESP) in China (Ma et al. 2013). In the past, this species was treated as the monotypic genus *Photinopteris* J.Sm. (Zhang and Gilbert 2013) but it was later transferred to *Aglaomorpha* (PPG I 2016) and now to *Drynaria* nom. consv. (Christenhusz and Schneider 2012).

In this study, we reported the complete chloroplast genome of *D. acuminata* for the first time. Fresh leaf material was collected from Xishuangbanna Tropical Botanical Garden, Chinese Academy of Science, Yunnan, China (N21°55′44″, E101°15′20″) and the voucher specimen was deposited at the Herbarium of Xishuangbanna Tropical Botanical Garden (HITBC, collection number: Liu-CP13). Genomic DNA was extracted from 2 g leaves using the CTAB method (Doyle and Dickson 1987), 0.5 µg DNA was fragmented to reconstruct short-insert (500 bp) libraries following the manufacturer’s manual (Illumina) and then used for sequencing. The DNA sample was indexed by tags and pooled together in one lane of a Genome Analyzer (Illumina HiSeq 2000) for sequencing at the Germplasm Bank of Wild Species, Kunming Institute of Botany (KIB) in Kunming, China. GetOrganelle toolkit (Jin et al. 2020) and Geneious (https://www.geneious.com) were employed to assemble and annotate the genome. CP genome of *Pyrrosia petiolosa* (MN885667) and *Drynaria roosii* (KY075853) was employed as reference genome The newly sequenced and annotated plastid genome was submitted to the GenBank (accession number MW042681).

The chloroplast genome of *D. acuminata* had the typical quadripartite structure; the total length is 151,591 bp including a large single-copy (LSC) region of 80,661 bp, a small single-copy (SSC) region of 21,688 bp, and a pair of inverted repeats (IR) regions of 24,621 bp. The chloroplast genome contains 131 genes including 88 protein-coding genes, 35 tRNAs, and 8 rRNA genes.

The chloroplast genome of *D. acuminata* was incorporated into a matrix including 23 taxa covering four subfamilies of Polypodiaceae (PPG I 2016) with the aim to reconstruct phylogenetic relationship of Polypodiaceae. 84 coding genes were selected, aligned, and concatenated into a single matrix using MAFFT (Katoh and Standley 2013). The phylogenetic analyses were constructed using IQ-tree with Maximum likelihood method (Minh et al. 2020), nucleotide substitution model of K-2-P was used with the 1000 bootstrap replicates. *Loxogramme lankokiensis* was selected as outgroup according to PPG I (2016).

In the phylogenetic tree, *D*. *acuminata* was grouped together with *D. roosii* with *Selliguea yakushimensis* and *Pecluma dulcis* formed a basal clade (Figure 1). Two subfamilies (Microsoroideae and Platycerioideae) were strongly supported as monophyletic and were found to be sister clades. Subfamily Polypodioideae was founded to be clustered with Drynarioideae. All *Pyrrosia* species were grouped together and formed a monophyletic clade.

**Figure 1.**
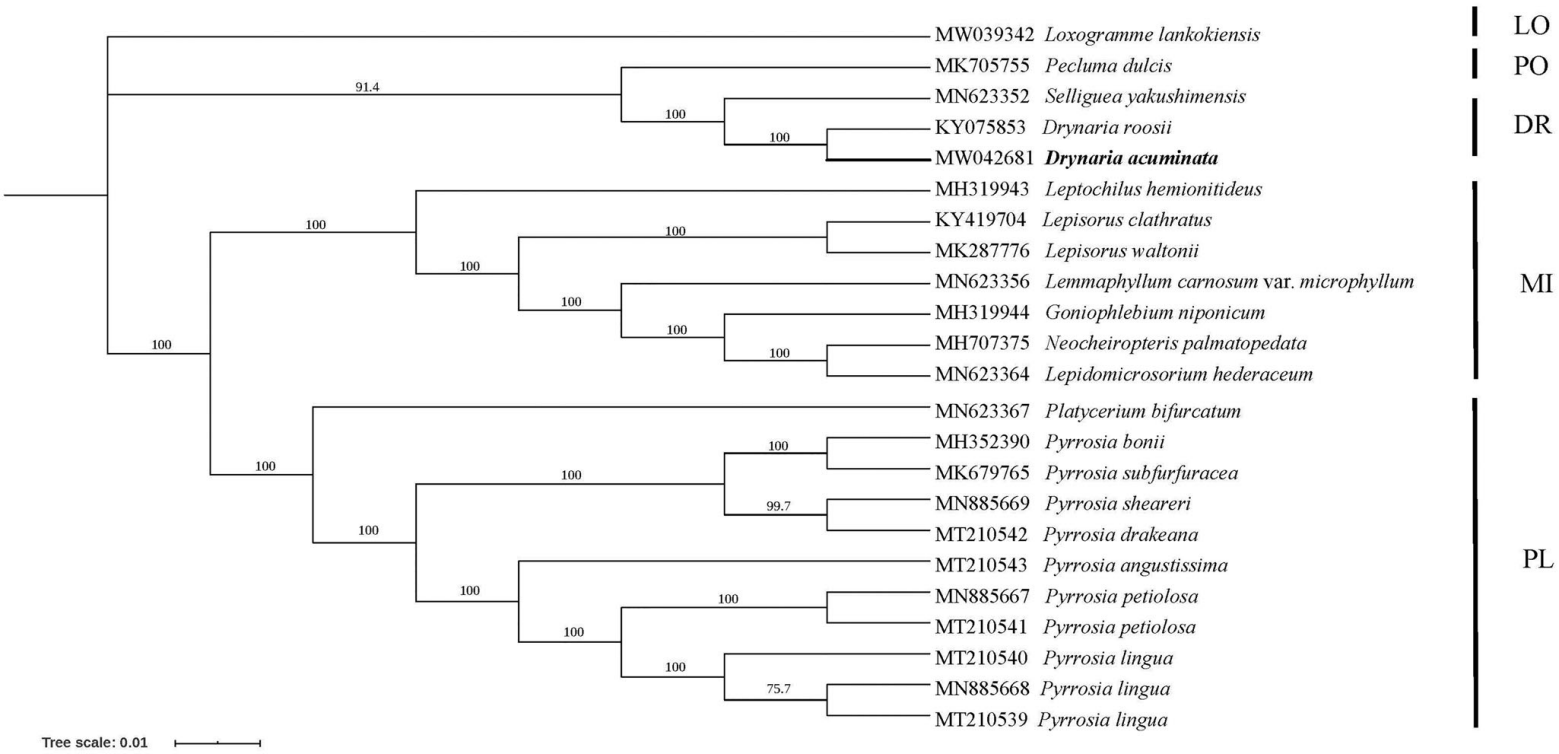
Maximum likelihood phylogeny reconstructed from 23 chloroplast genomes by IQ-tree. The sampling covered representatives of four subfamilies of Polypodiaceae. *Loxogramme lankokiensis* was selected as outgroup. DR: Drynarioideae; PO: Polypodioideae; MI: Microsoroideae; PL: Platycerioideae; LO: Loxogrammoideae.

The complete chloroplast genome sequence of *D. acuminata* will provide useful information for phylogeography of this tropical fern species and the phylogenomic study for the derived fern family Polypodiaceae.

## Data Availability

The data that support the findings of this research are openly available in GenBank of NCBI at https://www.ncbi.nlm.nih.gov, reference number MW042681.
